# High-Throughput Cancer Cell Sphere Formation for Characterizing the Efficacy of Photo Dynamic Therapy in 3D Cell Cultures

**DOI:** 10.1038/srep12175

**Published:** 2015-07-08

**Authors:** Yu-Chih Chen, Xia Lou, Zhixiong Zhang, Patrick Ingram, Euisik Yoon

**Affiliations:** 1 Department of Electrical Engineering and Computer Science, University of Michigan, 1301 Beal Avenue, Ann Arbor, MI 48109-2122; 2 Department of Biomedical Engineering, University of Michigan, 2200 Bonisteel, Blvd. Ann Arbor, MI 48109-2099, USA

## Abstract

Photodynamic therapy (PDT), wherein light sensitive non-toxic agents are locally and selectively activated using light, has emerged as an appealing alternative to traditional cancer chemotherapy. Yet to date, PDT efficacy has been mostly characterized using 2D cultures. Compared to 2D cultures, 3D sphere culture generates unique spatial distributions of nutrients and oxygen for the cells that better mimics the *in-vivo* conditions. Using a novel polyHEMA (non-adherent polymer) fabrication process, we developed a microfluidic sphere formation platform that can (1) generate 1,024 uniform (size variation <10%) cancer spheres within a 2 cm by 2 cm core area, (2) culture spheres for more than 2 weeks, and (3) allow the retrieval of spheres. Using the presented platform, we have successfully characterized the different responses in 2D and 3D cell culture to PDT. Furthermore, we investigated the treatment resistance effect in cancer cells induced by tumor associated fibroblasts (CAF). Although the CAFs can enhance the resistance to traditional chemotherapy agents, no significant difference in PDT was observed. The preliminary results suggest that the PDT can be an attractive alternative cancer therapy, which is less affected by the therapeutic resistance induced by cancer associated cells.

Photodynamic therapy (PDT) is a treatment that generates local oxidative stress to kill cancer cells upon illumination of light. Due to the capability to selectively activating the cytotoxicity in the target tumor region, it is known to have less side effects than conventional chemo-therapies^1^,^2^. There are three key factors that need to be characterized for effective PDT: the photosensitizer (PS), oxygen, and light^1^,^2^. During therapy, light is applied to activate the photosensitizer at a wavelength that corresponds to the photosensitizer’s maximum absorption. The excited photosensitizers transfer their energy to adjacent oxygen molecules to generate high energy oxygen molecules (singlet state oxygen) which in turn generate cytotoxic reactive oxygen species, causing the localized cytotoxicity^3^,^4^,^5^,^6^. As the efficacy of the PDT highly depends on these three elements, we previously developed an integrated microfluidic system that can comprehensively characterize and optimize PDT efficacy under different light, drug concentration, and oxygen conditions^7^. Within a core chip size of 5  mm by 5 mm, more than 1,000 PDT conditions could be simultaneously screened^7^,^8^. Although extensive combinatorial PDT conditions could be tested in the previous approach, it can only perform assays for a monolayer of cells in 2D culture, which poorly reflects the complexity of *in-vivo* environment^9^,^10^,^11^,^12^. Due to the unorganized and rapid growth of tumors, blood vessels often do not adequately supply oxygen and nutrients to the tumor microenvironement. This creates regions of low nutrition, low glucose, low pH, and low oxygen levels (hypoxia) within tumors. These conditions may boost drug resistance and induce mutation^9^,^10^. The conditions that prevent adequate supply of nutrients can also make it difficult for conventional drugs to permeate into these regions. As a result, the inability to eradicate the tumor cells in these regions of hypoxia can be a cause of tumor relapse. Thus, a good model that takes such factors into account is particularly important for drug screening in cancer. For PDT, which depends on photosensitizer concentrations and oxygen levels, it is critical to investigate the effect of drug efficacy in a 3D tumor environment. Compared to 2D monolayer cultures, 3D sphere culture better mimics drug and oxygen distribution in the tumor niche^11^,^12^.

There are a few approaches popular approaches to realize 3D sphere culture. Hanging drop method is one of the most popular approaches used for culture of 3D spheres^13^,^14^. One of the issues in the hanging drop approach is that cell culture environment is entirely exposed to the ambient environment, which may lead the evaporation of the media from the drops. The increase in osmolarity due to media concentration change is detrimental to cell viability; as a result, relatively large volumes (e.g. 10 μL) are used, limiting the minimum size of the drops^14^. Consequently, the number of hanging drops that can be deployed for a given area is relatively small. Moreover, media exchange is a challenge. Though some technical innovations have been implemented to facilitate media exchange^15^, it is generally necessary to manually pipette new media into each droplet individually, further limiting the number of spheres and their size scaling. There are other micro-fabricated approaches for large scale formation of spheres on open substrates, but it is difficult to identify and handle the formed spheres^16^,^17^,^18^,^19^. Forming spheres using micro-rotational flow or the magneto-Archimedes effect also has limitations in scalability^20^,^21^. Performing 3D culture in a hydrogel has been introduced, but the chemical and mechanical cues provided by the hydrogel can affect the behavior of spheres^22^,^23^,^24^.

Compared to these previous approaches, generating spheres within the enclosed microfluidic channels is attractive as evaporation is negligible and a smaller media volume (10–100 nL) can be used per sphere. Also, a single device inlet can supply media to all the enclosed microwells, facilitating simultaneous media exchange to all spheres by one pipetting operation. To create sphere culture environment in enclosed microchannels, surfactants (e.g. F-108), chemicals, or nano-structures were patterned into previous devices^25^,^26^,^27^,^28^,^29^,^30^,^31^,^32^. Although these methods can prevent cell adhesion for certain cell types, some highly adherent cells can still adhere on coated substrates, especially in the serum rich culture media which can contain many adhesion factors. Reliable non-adherent coatings are critical to avoid adhesion, which can alter the behavior of cells and prevent sphere formation. In this work, we present a microfluidic platform that allows for the high-throughput formation of cancer spheres on chip. 1,024 spheres can be reliably generated within the chip core area of 2 cm by 2 cm, and the formed spheres can be successfully cultured for more than 2 weeks. Using this platform, assays were performed to compare the PDT efficacy between the conventional 2D monolayer culture and 3D sphere culture. For T47D (breast cancer) cells, the IF_50_ (half maximal inhibitory fluence (light energy J/cm^2^) for 3D spheres is by four times higher than that for 2D monolayer culture, verifying the enhancement of PDT resistance in spheres due to low photosensitizer permeation and low oxygen levels.

We also investigated the therapeutic resistance of tumor cells induced by supporting somatic cells. Fibroblasts are a major micro-environmental regulator in cancer and play an important role in tumorigenesis, drug resistance, and metastasis^33^,^34^,^35^,^36^. Recently, it was reported that the co-culture of cancer cells and fibroblast can boost chemotherapeutic resistance^35^,^36^. However, as the previous 3D PDT work provide poor microenvironment control and low throughput^37^, whether or not the same effects are observed in PDT has not yet been fully explored. Here, we performed a simple experiment to observe whether fibroblast conditioned media (CM) can boost the resistance to PDT in cancer spheres. We used cisplatin (chemo-therapy drug) as a control because it shows a significant reduction in its efficacy when cells exposed to fibroblast CM, As compared with cisplatin, we found that the PDT efficacy was not affected by fibroblast CM, suggesting it is less affected by cancer associated cells.

## Results

### High-throughput sphere formation

**Figure 1:**
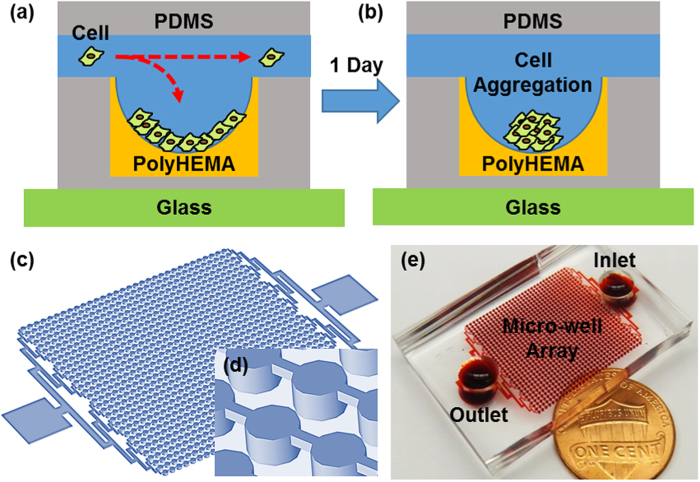
High-throughput sphere formation microfluidic chip. (**a**) Schematic showing cells settling into a microwell during loading. (**b**) Schematic showing cell aggregation and sphere formation 1 day after cell loading. (**c**) 3D schematic view of the microfluidic chip. (**d**) Enlarged 3D schematic of micro-wells. (**e**) The fabricated device that contains 1,024 micro-wells within a core area of 2 cm by 2 cm. The cells are loaded in the inlet (top right) and then flow through the microwell array to the outlet (bottom left).

The presented high-throughput sphere formation platform is composed of an array of 1,024 non-adherent microwells connected by single inlet and outlet (Fig. 1). Spheres are formed by aggregation of cells in each microwell (400 μm in depth) after cell loading (Fig. 1). When cells are loaded into the channel, a portion of the cells will settle down into a microwell and the rest will flow into the rest of downstream microwells (Fig. 1(a)). As the number of cells that settle in a well depends on the size of micro-well, we can easily generate larger and smaller spheres for different applications by changing the diameter of microwells. In our study, we used two different microwell diameters, 250 μm and 450 μm, respectively. To facilitate sphere formation by cell aggregation, micro-wells were coated with polyHEMA. PolyHEMA is a biocompatible hydrogel that has been used as a non-adherent coating material for suspension cell culture successfully for more than 30 years^38^,^39^. As cells cannot adhere onto the polyHEMA-coated layer, they aggregate to form a sphere (Fig. 1(b)). Using the presented device (Fig. 1(c–e)), we can reliably generate 1,024 spheres at high throughput within 1 day.

### PolyHEMA coating process

**Figure 2:**
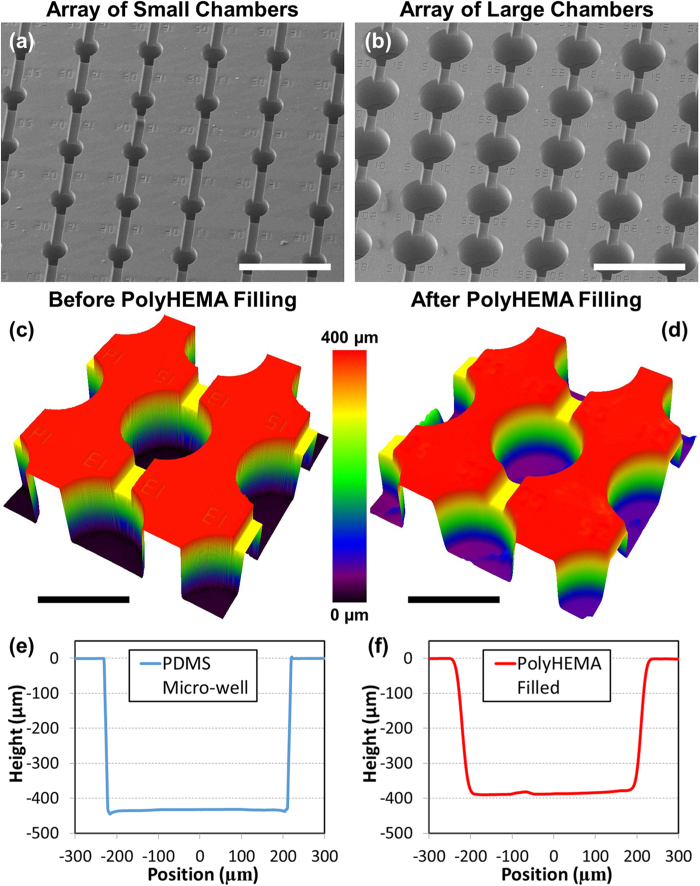
Fabrication of the non-adherent microwells. (**a, b**) SEM images of an array of microwells of small (250 μm in diameter) and large (450 μm in diameter) chamber sizes (scale bar: 1 mm). (**c, d**) 3D profile of a large microwell before and after polyHEMA coating from a LEXT microscope (scale bar: 500 μm).(**e, f**) Cross-sectional profiles of a large microwell before and after polyHEMA coating. PolyHEMA thickness at the center was measured as 40 μm.

Our platform was fabricated by stacking two layers of PDMS. The first PDMS layer (substrate layer) has microfluidic channels and microwells coated with polyHEMA to avoid cell adhesion. The second PDMS layer (top layer) is an unpatterned cap (blank layer) for enclosure of the channel layer. These two PDMS layers (blank layer and substrate layer) were fabricated using standard soft lithography processes separately and then bonded after plasma oxygenation as shown in Fig. S1. For the substrate layer, two masks were used to form a SU8 (Microchem) master mold: the first mask for the microfluidic channels (100 μm in height) and the second mask for the microwells (400 μm in height). An unpatterned master was used to fabricate the blank layer. The fabricated substrate layer with both smaller (250 μm) and larger (450 μm) microwells are shown in Fig. 2 (a,b).

To make the microwells non-adherent, polyHEMA was filled in the suspension culture chambers using a stamping process developed in our lab from previous work^40^. The polyHEMA was dissolved in an ethanol solution (60 mg/mL in 95% ethanol) and applied on the substrate PDMS layer. A piece of blank PDMS was pressed on top to squeeze out any excess solution, leaving the polyHEMA only in the indented micro-wells. To improve the coating quality, the indented PDMS substrate was plasma treated to increase its hydrophilicity. Because polyHEMA has higher affinity to hydrophilic substrates, the treatment increases the polyHEMA adhesion to the patterned PDMS substrate while stamping. After that, the substrate layer and the blank PDMS stamp were put on a hot plate at 110 °C for 2 hours under pressure (~13 k Pa), in order to facilitate evaporation of ethanol through the PDMS. As ethanol evaporates from the solution, polyHEMA is condensed and coated onto the suspension cell-culture microwells. To remove any residual polyHEMA deposited outside the microwells, which can affect the bonding strength between two layers of PDMS, 30 seconds of 800 Watt plasma etching was performed using a YES polymer striper (the estimated etching depth of 0.3 μm). This process ensures clean PDMS surface with polyHEMA coated only on the suspension culture microwells.

### Characterization of polyHEMA-coated surface

In order to verify successful polyHEMA microwell deposition, we examined the surface profile before and after polyHEMA coating. The surface was scanned using a laser interference microscope (LEXT, Olympus), as shown in Fig. 2 (c,d). Before polyHEMA coating, the vertical sidewall of the indented microwell was clearly visible. After polyHEMA filling, the slope of sidewall became smoother, indicating that polyHEMA was coated in the well. Based on the cross-sectional profile of microwells before and after polyHEMA coating (Fig. 2(e,f)), the coating depth at the center of the microwell is measured approximately 40 μm, which is sufficient to provide reliable a non-adherent culture surface. Additionally, the rounding of the substrate profile is beneficial as it promotes cell aggregation in the well.

To verify non-adherent culture in the microwells, T47D breast cancer cells were loaded and cultured on a polyHEMA-coated microwell array. We used an uncoated PDMS microwell array (400 μm deep) as a control. In the uncoated wells, the cells adhered not only inside the wells but also on the sidewall and outside of the wells (Fig. S2(a)). In contrast, we can clearly observed that cells do not adhered inside the wells in the polyHEMA patterned array. Cells were only able to adhere on the top surface outside the well where the polyHEMA was removed via stamping and plasma etching (Fig. S2(b)). Multiple cell lines including MCF-7 (breast cancer) and SUM159 (breast cancer) cells were tested on the substrate in addition to the T47D cells. Non-adherent culture was observed in all these cell lines, indicating that the polyHEMA coating process is robust and reliable.

### Sphere formation assays

**Figure 3:**
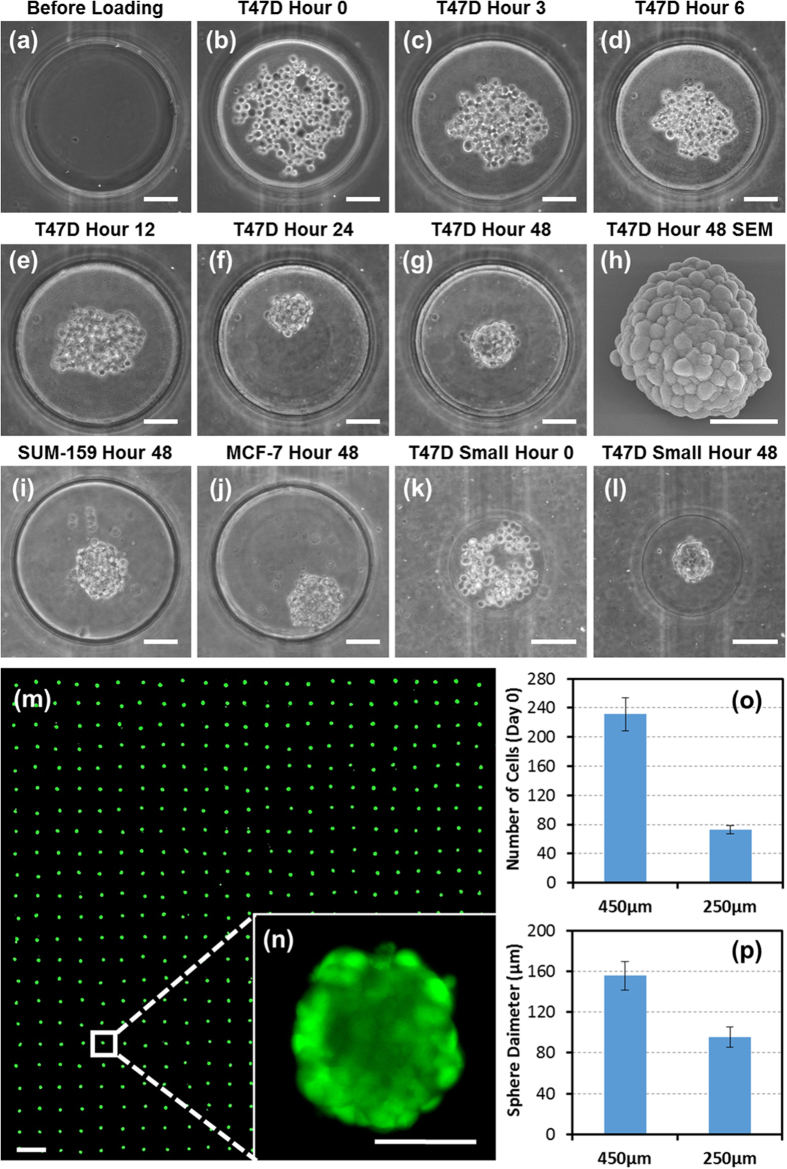
Characterization of sphere formation on chip. (**a-g**) Sphere formation of T47D breast cancer cells in a large microwell: (**a**) before cell loading, (**b**) right after cell loading, (**c**) 3 hours, (**d**) 6 hours, (**e**) 12 hours, (**f**) 24 hours, and (**g**) 48 hours after cell loading. (scale bar: 100 μm) (**h**) SEM image of a sphere retrieved from the chip after 48 hour culture. (scale bar: 50 μm) (**i**) A sphere formed by SUM-159 breast cancer cells after 48 hours. (scale bar: 100 μm) (**j**) A sphere formed by MCF-7 breast cancer cells after 48 hours. (scale bar: 100 μm) (**k–l**) Sphere formation of T47D breast cancer cells in a small microwell: (**k**) right after cell loading and (**l**) 48 hours after cell loading. (scale bar: 100 μm) (**m**) An array of spheres formed in the device. The spheres were stained with green Cell Tracker. (scale bar: 1 mm) (**n**) An enlarged view of a sphere in a microwell. (scale bar: 100 μm) (**o**) Average number of cells loaded in the large and small microwells. (N = 20 micro-wells) (**p**) Average diameter of spheres in the large and small microwells. (N = 20 spheres).

After characterization of the polyHEMA-coated substrate, we performed sphere formation experiments. To guarantee that a consistent number of cells are loaded into each microwell, we applied a high flow rate (300 μL per minute) at a high concentration (5 × 10^6^ cells per mL) of T47D cells during cell loading^31^. As only a small portion (~5%) of loaded cells settled down in the wells, similar numbers of cells were present in the upstream as well as downstream microwells. Thus, there is no significant difference in the number of settled cells per well between the upstream and downstream (Fig. S3). Right after cell loading, cells were uniformly distributed inside each microwell as shown in the Fig. 3(b). The loaded cells aggregate into spheres at the center due to non-adherent coating of polyHEMA and rounded substrate profile. After 3 hours, we could clearly observe preliminary cell aggregation (Fig. 3 (c)). As time goes by, the aggregation became tighter and tighter (Fig. 3(d–f)), and compact spheres were observed after one day of culture. After culturing for 2 days, we peeled off the top PDMS layer to retrieve the spheres. The process did not damage the spheres, and we could successfully collect the spheres for other downstream analyses (Fig. S4). A retrieved sphere is shown under SEM (Fig. 3 (h)). Additionally, we demonstrated long term sphere culture for more than 2 weeks using T47D cells (Fig. S5).

Using the same process, we also formed spheres from MCF-7 and SUM159 breast cancer cells (Fig. 3 (i,j)), demonstrating that the presented platform can culture various cell lines reliably. In addition, sphere size can be controlled by modifying the size of the microwell without changing the protocol. This is because more cells settled in a larger well than a smaller one (Fig. S6). The sphere formation process in a small well (250 μm in diameter) is shown in Fig. 3 (k,l). Figure 3 (m) shows an array of fluorescently labelled (Cell Tracker, C2527, Life Technology) spheres formed in the platform, and the enlarged view of a fluorescently labelled sphere is shown in the Fig. 3 (n). Since the standard deviation of loaded cells is less than 10% of the average number of loaded cells in each microwell (Fig. 3 (o)), we can achieve uniform sphere formation throughout the whole array (Fig. 3 (p)).

### Difference in PDT efficacy between 2D and 3D cell cultures

**Figure 4:**
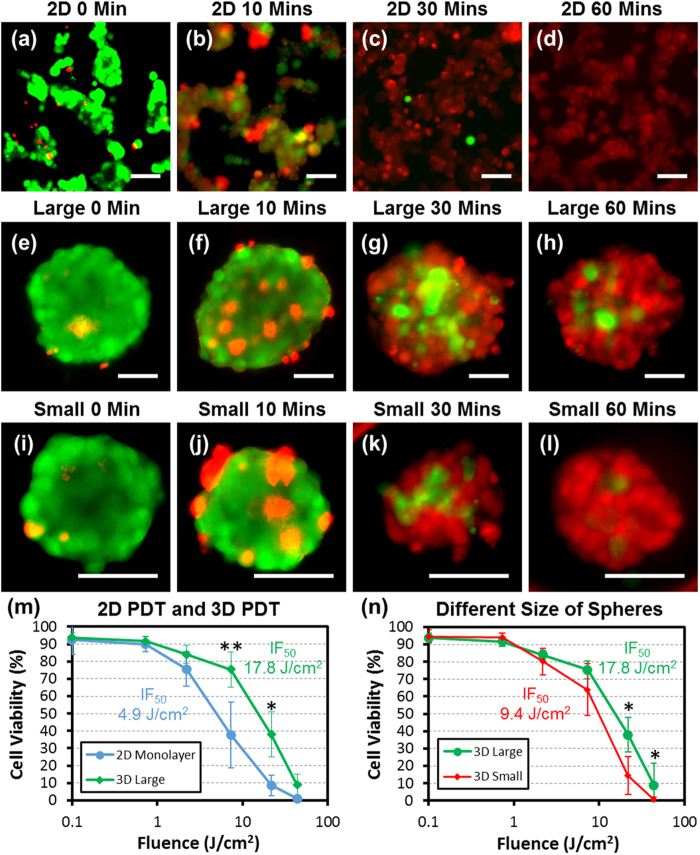
Differences in efficacy of PDT between 2D monolayer cell cultures and 3D spheres. (**a-d**) Images of T47D 2D cell cultures after PDT with LIVE (green) /DEAD (red) staining. 10 μM methylene blue in PBS was used as photosensitizer with light exposure doses of (**a**) 0 minutes, (**b**) 10 minutes, (**c**) 30 minutes, and (**d**) 60 minutes. The fluence per minute is 0.73 J/cm^2^. (scale bar: 50 μm) (**e-h**) Images of large (average diameter: 155 μm) T47D spheres after PDT with LIVE (green) /DEAD (red) staining. Light exposure doses of (**e**) 0 minutes, (**f**) 10 minutes, (**g**) 30 minutes, and (**h**) 60 minutes were used. (scale bar: 50 μm) (**i-l**) Images of small (average diameter: 95 μm) T47D spheres after PDT with LIVE (green) /DEAD (red) staining. Light exposure doses of (i) 0 minutes, (**j**) 10 minutes, (**k**) 30 minutes, and (l) 60 minutes were used. (scale bar: 50 μm) (**m**) Differences in PDT efficacy between T47D spheres and 2D monolayer culture with different light doses (fluence). The IF_50_ (half maximal inhibitory fluence) of 2D cell monolayer culture is estimated to be 4.9 J/cm^2^ (N = 5 cultures), while the IF_50_ of large spheres is estimated to be 17.8 J/cm^2^ (N = 10 spheres) (n) PDT efficacy differences as a function of fluence for large and small spheres. The IF_50_ of small spheres (average diameter: 95 μm) is estimated to be 9.4 J/cm^2^. (N =  10 spheres) The IF_50_ of large spheres (average diameter: 155 μm) is estimated to be 17.8 J/cm^2^. (N = 10 spheres) * refers to P < 0.05, and ** refers to P < 0.01.

After characterization of sphere formation in the platform, we performed photodynamic therapy (PDT), comparing conventional 2D mono-layer cell cultures and 3D cell spheres. Based on our previous work^7^, we chose to use a 10 μM methylene blue (photosensitizer) solution and ambient oxygen level (20%) for treatment of T47D cells. After incubation with the drug for 1 hour, 2D and 3D cultures were illuminated with various exposure doses, from 14 seconds (0.1 J/cm^2^) to 1 hour (total 43.8 J/cm^2^), for the activation of photosensitizers. Right after exposure, we performed LIVE/DEAD staining to evaluate cell viability. Figure 4 shows the distinct difference between the 2D and 3D PDT responses. Under an exposure of 7.3 J/cm^2^, around 50% of the cells were killed in the 2D cell monolayer (Fig. 4 (b)), while at the same dose, most of the cells were viable in the 3D spheres (Fig. 4(f)). When the illuminating exposure is 21.9 J/cm^2^, almost all the cells in the 2D monolayer were killed (Fig. 4(c)), but many cells in the sphere were still viable (Fig. 4(g)). Even when the light exposure is as high as 43.8 J/cm^2^, still a portion of cells at the center of spheres were shown to be viable (Fig. 4(h)). As expected, the size of sphere also affects the efficacy of the PDT. Larger spheres (Fig. 4 (e–h)) are more resistant than the small spheres (Fig. 4(i–l)) under the same PDT treatment conditions. The cell viabilities vs. light exposure (fluence) under the different conditions are plotted in Fig. 4 (m,n). To quantify the efficacy of PDT, IF_50_ (half maximal inhibitory fluence) was defined as the counterpart of IC_50_. From these plots, the IF_50_ of the 2D monolayer is estimated to be 4.9 J/cm^2^, while the IF_50_ of small and large spheres are estimated to be 9.4 J/cm^2^ and 17.8 J/cm^2^, respectively. The results clearly show the enhancement of PDT resistance in sphere culture.

### Drug resistance enhancement effect caused by fibroblast condition media

**Figure 5:**
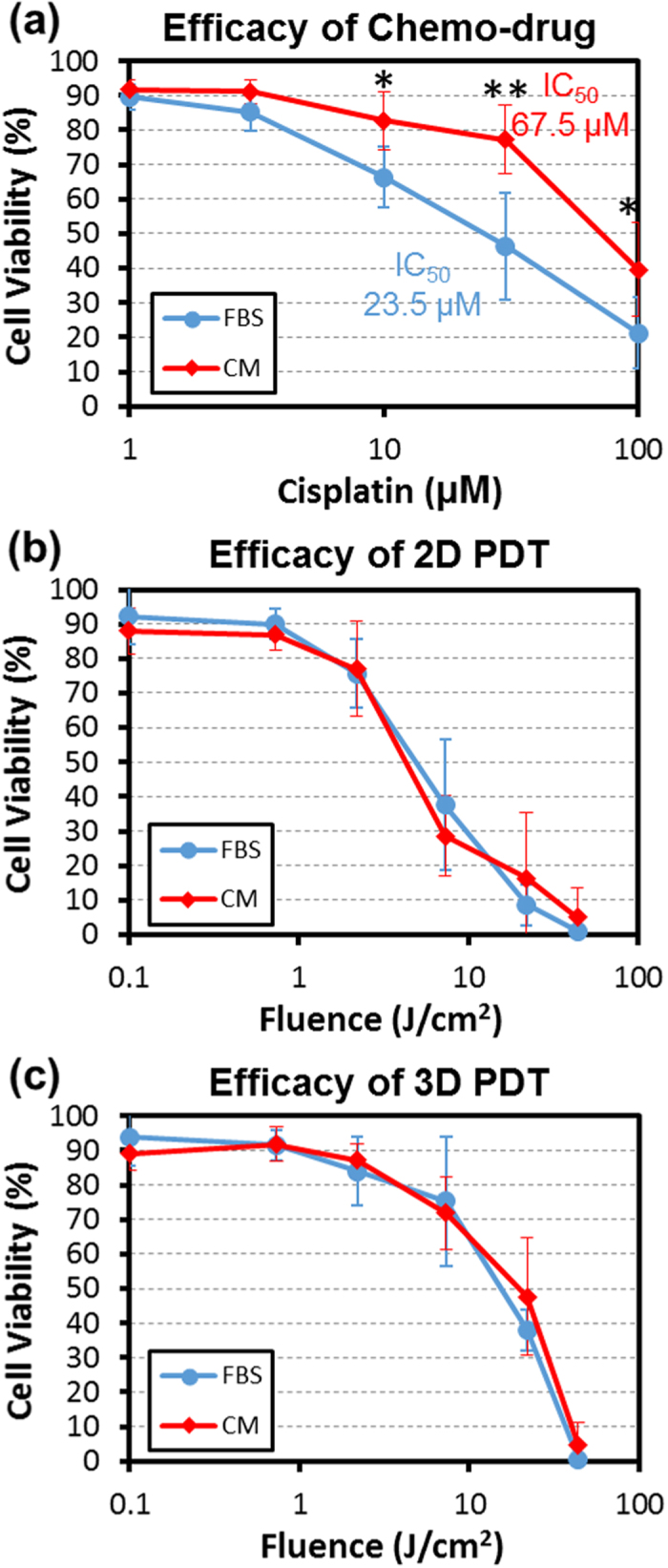
Effect of fibroblast conditioned media on chemotherapy and PDT efficacy. (**a**) Efficacy of cisplatin treatment on T47D cell monolayer cultures. When the T47D cells were cultured with media + FBS rather than conditioned media (CM), the IC50 was estimated to be 23.5 μM. However, with fibroblast CM, the IC50 increased to 67.5 μM (N = 5 cultures), verifying that the fibroblast conditioned media can boost chemodrug resistance. (**b**) Efficacy of PDT on 2D monolayer cell cultures with and without fibroblast conditioned media. No significant difference was observed between the two culture conditions. **(c**) The efficacy of PDT on 3D large spheres with and without the fibroblast conditioned media. No significant difference was observed between the two culture conditions. * refers to P < 0.05, and ** refers to P < 0.01.

This robust, high throughput sphere formation device is useful in a variety of other screens. It has been reported that co-culture of cancer cells and fibroblasts can boost drug resistance of the cancer cells to conventional chemotherapies^35^,^36^. This implies that it would be more difficult to treat cancer cells in the *in-vivo* cancer niche as compared to *in-vitro* culture. However, it has not been studied whether the efficacy of PDT on cancer cells would be degraded by the influence of fibroblast. In this work, we used the media conditioned by the secretion from cancer associated fibroblast to mimic the influence of fibroblast cells in the cancer niche. In Fig. 5 (a) and Fig. S7 it was confirmed that cisplatin treatment (a conventional chemotherapy) of T47D cells becomes less effective when cultured in fibroblast conditioned media (CM). The cells cultured in the normal serum-containing media have an IC_50_ of 23.5 μM, while the cells cultured in CM have an IC_50_ of 67.5 μM, verifying the effect of fibroblast induced drug resistance^41^,^42^,^43^. Under the same culture conditions, PDT was evaluated. We observed no significant increase in PDT resistance when comparing CM to normal culture media (2D culture in Figs 5 (b) and 3D culture in Fig. 5 (c), respectively), indicating that fibroblast mediated resistance does not boost resistance to PDT.

## Discussion

3D sphere cell culture has emerged as a better model for drug screening, as 2D monolayer cell culture does not adequately mimic *in-vivo* environments. However, the formation and handling of 3D cell spheres is less reliable in current platforms, and 3D culture techniques have not yet been widely adopted^11^,^12^. The hanging drop method, one of mostly used approaches, has drawbacks in scaling down droplet sizes for high-throughput and difficulty in media exchange during culture. Our presented platform has overcome these drawbacks by realizing non-adherent sphere formation inside enclosed microfluidic channels. Selective polyHEMA coating in each microwell facilitates reliable aggregation of loaded cells for sphere formation within 24 hours. In our 3D culture platform, we have achieved: (1) reliable formation of cancer spheres, (2) high sphere formation yield (over 95%), (3) uniform sphere size (size variation less than 10%), (4) capability to culture spheres for more than 2 weeks, and (5) capability to retrieve spheres from the device for further analyses. Thus, the presented platform is useful for high-throughput therapeutic screening on 3D cell spheres.

Using our platform, we can easily investigate the difference in PDT efficacy between 2D cell cultures and 3D cell spheres. We applied the selective coating of polyHEMA for 3D sphere culture while using the uncoated devices for 2D adherent culture as controls. In 2D monolayer cell culture, all cells are exposed to a uniform photosensitizer dosage and same oxygen level. However in 3D spheres, photosensitizer and oxygen need to diffuse into the center, decreasing photosensitizer and oxygen levels in the middle of spheres. As PDT relies on excitation of available photosensitizer concentrations and reactive oxygen species, the depletion of photosensitizer and oxygen at the center of the sphere can degrade the efficacy of PDT^3^,^4^,^5^,^6^,^7^. In addition, the cells at the center are supported by more surrounding cells, and these interactions can enhance the cell survival^44^,^45^. In literature, it was reported that the necrosis can happen at the center of sphere due to the lack of nutrition and oxygen^11^,^12^. However, because of the small sphere size we made and short culture time, we did not observe this phenomenon. We could clearly observe a higher survival rate at the center of spheres. Even after 60 minutes of light exposure, which completely killed cells in the 2D cultures, some cells at the center of the spheres were still viable. Overall, the IF_50_ of 3D spheres is by four times higher than that of 2D cultures. This apparent difference in drug response between 2D and 3D cell cultures highlights the importance of the 3D sphere models for more closely mimicking cancer niche behavior during pre-clinical drug screening.

The interaction between cancer cells and surrounding support cells also plays a critical role in influencing niche behavior. It is believed that cancer cells influence the micro-environment and exploit support cells to assist tumorigenesis, metastasis, and drug resistance^33^,^34^,^35^,^36^. This deteriorates effectiveness and makes the efficacy of cancer treatment *in-vivo* very unpredictable. Among many different types of supporting cells, we focused on cancer associated fibroblast cells^33^. In order to investigate their effect on drug and PDT efficacy, we used the conditioned media from the cancer associated fibroblast cells. These cells were harvested from a mouse xenograft and interacted with tumor cells for four weeks *in-vivo*. As expected, the conditioned media boosted cisplatin drug resistance in the treated cancer cells. The IC_50_ of the cells cultured in CM is almost three times higher than that of the cells cultured in the normal media. In contrast, we did not observe any significant difference in PDT under CM conditions. For both 2D and 3D cultures, the efficacy of PDT was not affected by the fibroblast CM. This may be due to the result of different mechanisms of cytotoxicity between chemo drugs (cisplatin) and PDT. For cisplatin, the primary mechanism relies on damaging the DNA to induce apoptosis^41^. However, cells may evade this mechanism by up-regulating DNA repair mechanisms and inhibiting apoptotic pathways. The cancer associated fibroblast cells influence cancer cells by enhancing these drug resistance mechanisms^35^,^36^. In comparison, the damage caused by PDT is far more diversified. The excited photosensitizers can react with oxygen to generate highly reactive radicals, which initiate the radical chain reactions damaging DNA, mitochondria, and lysosomes, and causing the depolarization of cell membranes^3^,^4^,^5^,^6^. This results in necrotic cell death rather than apoptosis. These wide spread damages cannot be easily mitigated by up-regulation of certain pathways, and this may explain the reason why we did not observe distinct resistance to PDT under the fibroblast conditioned media culture. Fortunately, it is likely that the PDT will kill both cancer cells and the tumor associated support cells *in-vivo* during therapy. As such, PDT can be an attractive alternative to conventional chemo-therapies.

## Methods

### Cell culture

T47D (breast cancer), SUM-159 (breast cancer), and MCF-7 (breast cancer) cells were obtained from Dr. Max Wicha’s lab (University of Michigan, MI, USA). T47D cells (breast cancer) were cultured in RPMI (Gibco 11875) with 10% FBS (Gibco 10082) and 1% penicillin/streptomycin (Gibco 15140). SUM159 and MCF-7 cells were cultured in DMEM (Gibco 11965) with 10% FBS (Gibco 10082) and 1% penicillin/streptomycin (Gibco 15140).

### Harvest cancer associated fibroblast cells and prepare fibroblast conditioned media

The primary fibroblast cells were obtained from Dr. Max Wicha’s lab. First, primary tumor tissue was obtained from mouse xenografts, and then minced into fine pieces using two scalpels. The minced tissue was transferred into a 50 mL conical tube, where Collagenase in a 1:1 ratio was added. The tissue was then gently dissociated on a rotary shaker (200 RPM) for 30–60 minutes or until all larger tissue fragments were digested. After dissociation, the tube was centrifuged at 40 x g for 2 minutes. Then, the supernatant was pipetted carefully into a new 50 mL conical tube. The fibroblasts were spun down at 250 x g for 5 minutes. After spinning down, the pellet was washed with HBSS and spun down again using 250 x g for 5 minutes. After centrifugation, the pellet was re-suspended in fibroblast media and plated in a cell culture flask. The media will be changed after 1.5 hours. The collected cancer associated fibroblasts were expanded in fibroblast media in an incubator at 37 °C and 5% CO_2_. To obtain fibroblast condition media, 10^6^ cancer associated fibroblasts were first cultured in a 60 mm diameter dish. After two days, the dish was washed by 2 mL PBS three times, and then 2mL of fresh media was added to the dish. After 48 hours, the supernatant was retrieved and centrifuged at 300 x g for 10 minutes to remove any cell debris.

### Cell loading for sphere formation

Before cell loading the device was pre-treated with 1% (w/v) Pluronic F108 solution to prevent nonspecific adhesion of the cells to the PDMS sidewalls while loading. For 2D cell monolayer culture, collagen (Collagen Type 1, 354236, BD Biosciences) was coated in the microwell to improve cell adhesion on the PDMS layer. After the devices were prepared, cells were harvested from culture plates with 0.05% Trypsin/EDTA (Gibco 25200) and then centrifuged at 1,000 rpm for 5 min. To improve image quality, cells can be stained with red fluorescent (Invitrogen, Cell tracker C2927) or green fluorescent (Invitrogen, Cell tracker Green C2925) dye before loading. The cells were then re-suspended in culture media to a concentration of 5 × 10^6^ cells/ml. 300 μL of this cell solution was flowed through the device within one minute, and then the cell solution in the inlet was replaced with fresh cell culture media.

### PDT experiments

One day after the cell loading, the cells adhered (for 2D) or aggregated (for 3D) in each microwell. For PDT assays, 200 μL of PBS was added into the inlet to wash the media from the device for 10 minutes. Then, 200 μL of 10 μM methylene blue (319112, Sigma-Aldrich) in PBS was pipetted into the inlet. The device was incubated for one hour with the methylene blue solution. Then, PBS was perfused for 10 minutes to wash away the methylene blue solution. The device was then placed in a 100 mm diameter dish to prevent media evaporation and was exposed under a red LED light source (Philips Lumileds LUXEON® Z Red (LXZ1-PD01) LED, peak emission 627nm, intensity 0.73 J/cm2/min Fig. S8). Although PDMS absorbs ~10–20% of light intensity, the attenuation is identical in both 2D and 3D PDT devices. After the light exposure, LIVE/DEAD staining (L3224, Life Technologies) was performed to assess cell viability. Calcein AM (green/Life) was diluted to 2 μM in PBS, and Ethidium homodimer-1 (red/dead) was diluted to 2 μM in PBS. The cell viability of 2D cultures was measured by counting red/green cells under fluorescent microscope, and the cell viability of 3D spheres was measured by counting red/green cells in the focal planes of every 20 μm. Three experiments were performed in independent devices for each data point. The methylene blue alone, light alone, and sham controls data are listed in the Table S1.

### Cisplatin drug treatment

After 1 day of culture, 200 μL of PBS was added into the inlet to wash away the media in the device for 10 minutes. Then, the 200 μL of cisplatin (various concentrations) in culture media was pipetted into the inlet. The media was changed daily with a fresh solution of the drug. After 3 days of treatment, LIVE/DEAD staining (L3224, Life Technologies) was performed to assess cell viability. Calcein AM (green/Life) was diluted to 2 μM in PBS, and Ethidium homodimer-1 (red/dead) was diluted to 2 μM in PBS. The cell viability of 2D cultures was measured by counting red/green cells under fluorescent microscope.

### Fibroblast conditioned media experiments

For the experiments testing the effect of fibroblast conditioned media, the conditioned media was used to replace culture media right after cell loading. For the cisplatin drug treatment experiments, the cisplatin was diluted in the conditioned media for the entire 3 day treatment.

### Statistical Analysis

One way ANOVA (SPSS) tests were used for all comparisons with a significance level of 0.05, considered statistically significant. Results are presented as mean ± SD.

## Additional Information

**How to cite this article**: Chen, Y.-C. *et al.* High-Throughput Cancer Cell Sphere Formation for Characterizing the Efficacy of Photo Dynamic Therapy in 3D Cell Cultures. *Sci. Rep.* **5**, 12175; doi: 10.1038/srep12175 (2015).

## Supplementary Material

Supporting Information
