# Supplementary material for: High-Throughput Cancer Cell Sphere Formation for Characterizing the Efficacy of Photo Dynamic Therapy in 3D Cell Cultures
Source: Sci Rep. 2015 Jul 8;5:12175. doi: 10.1038/srep12175 (PMC4495468; doi:10.1038/srep12175)
Supplement: Supporting Information [file srep12175-s1.pdf]

Title: High-Throughput Cancer Cell Sphere Formation for Characterizing the Efficacy of Photo Dynamic Therapy in 3D Cell Cultures

Authors and affiliations:

Yu-Chih Chen[a^], Xia Lou[a^], Zhixiong Zhang[a], Patrick Ingram[b], and Euisik Yoon*[a, b]

[a]Department of Electrical Engineering and Computer Science, University of Michigan, 1301 Beal Avenue, Ann Arbor, MI 48109-2122;

[b]Department of Biomedical Engineering, University of Michigan, 2200 Bonisteel, Blvd. Ann Arbor, MI 48109-2099, USA

^Authors made equal contributions

*Corresponding author

Yu-Chih Chen
1301 Beal Avenue, Ann Arbor, MI 48109-2122, USA
Tel: 734-272-7113; E-mail: yuchchen@umich.edu.

Euisik Yoon
1301 Beal Avenue, Ann Arbor, MI 48109-2122, USA
Tel: 734-615-4469; E-mail: esyoon@umich.edu.

Supporting Information

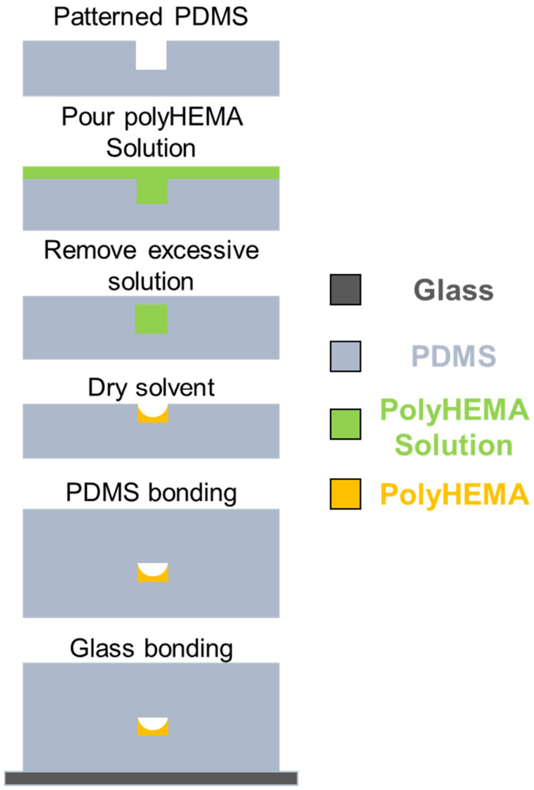

Fig. S1. Device fabrication processes. The device is composed of two PDMS layers. The first layer (substrate layer) has microfluidic channels and microwells coated with polyHEMA to avoid cell adhesion. The second layer (blank cover layer) is used to enclose the channels in the substrate layer. These two PDMS layers (substrate and substrate layers) were fabricated using standard soft lithography processes separately and then bonded via plasma oxygenation.

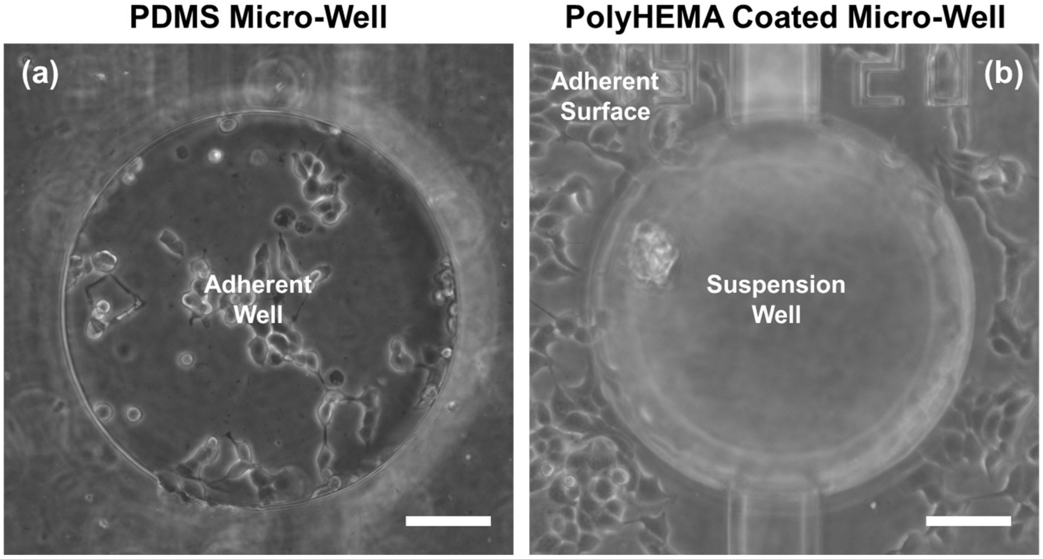

Fig. S2. Test of selectivity in cell adhesion without and with polyHEMA coating. (a) Without polyHEMA coating, we observed that cells adhere in and around the microwell. (b) With polyHEMA coating inside the microwell, cells can only adhere outside, but not inside the microwell.

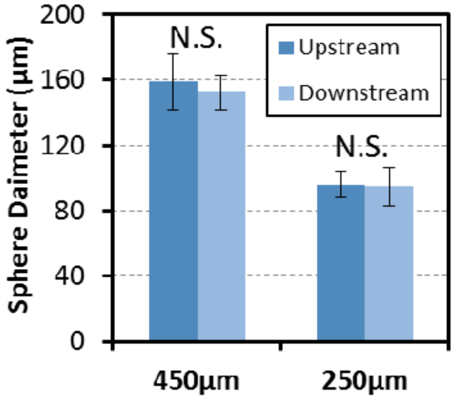

Fig. S3. Average size of spheres in upstream and downstream microwells in an array of 1,024 microwells. No significant difference was observed both in the large (450 µm) and small (250 µm) microwells demonstrating uniform-size spheres can formed across the entire device.

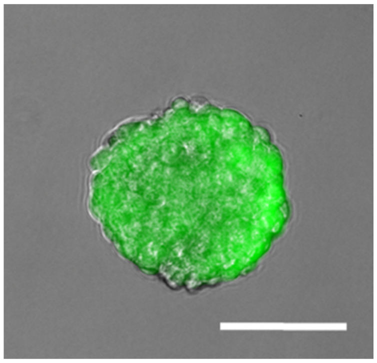

Fig. S4. A sphere retrieved from the device after 48 hours of culture. (scale bar: 100 μm)

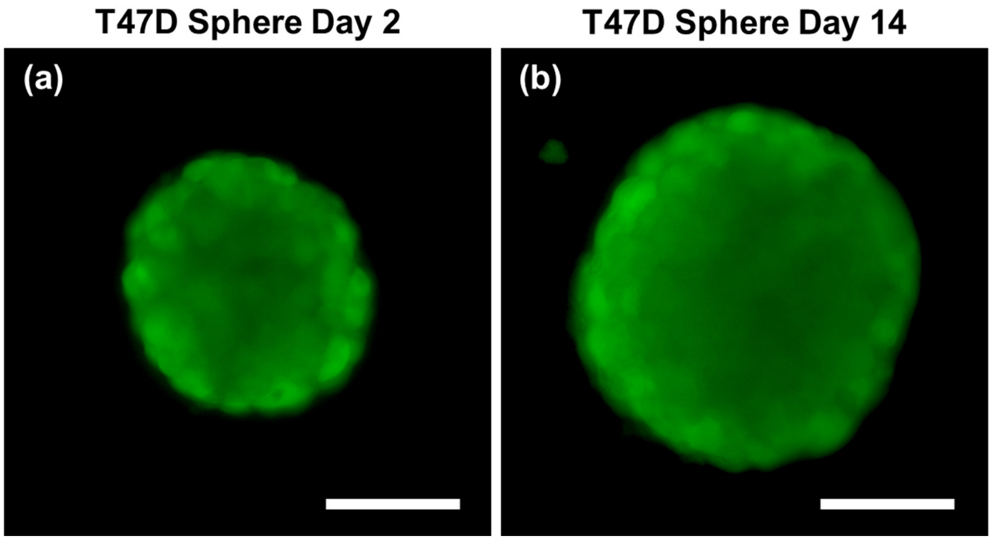

Fig. S5. Long term sphere culture on chip. (a) A sphere on day 2 and (b) day 14. (scale bar: 100 µm)

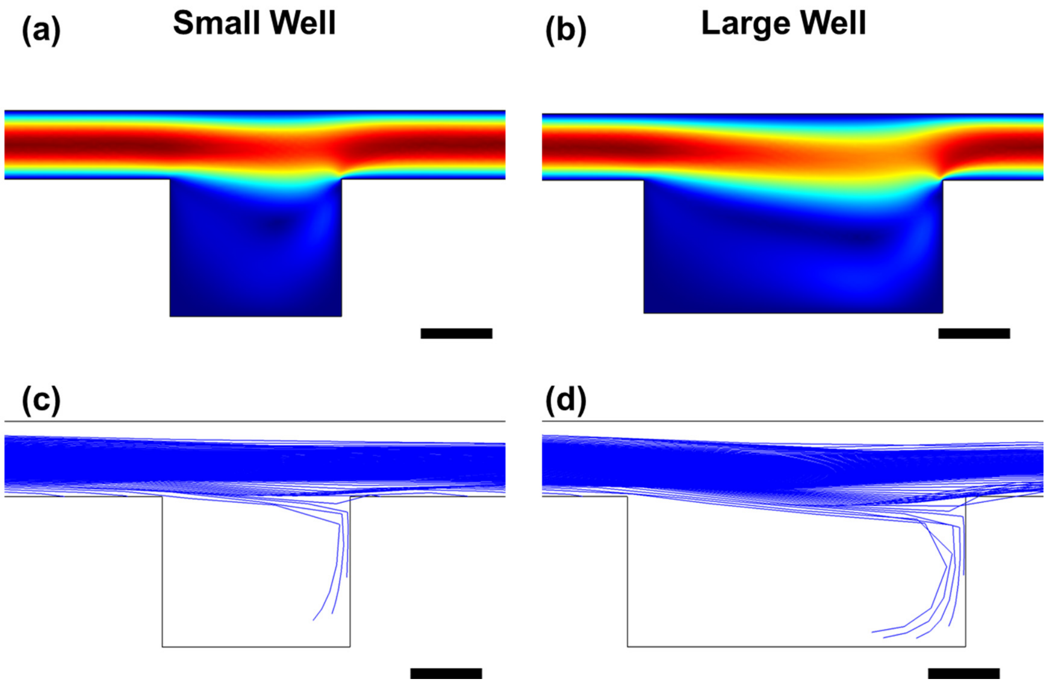

Fig. S6. COMSOL simulations of particle trajectory in small (250 µm) and large (450 µm) microwells. Cells were modeled as particles with 10 µm in diameter with $1.1X10^3$ kg/m^3 in density. Flow rate was set to 300 µL per minute (comparable to actual flow on chip). 1,000 particles were introduced from the inlet and traced. (A) Flow velocity field of in a small microwell. (B) Flow velocity field of in a large microwell. (C) Trajectories of cells in a small microwell. (D) Trajectories of cells in a large microwell. More cells are captured in a large well than a small well. In this manner, the size of a sphere can be controlled by the size of microwells.

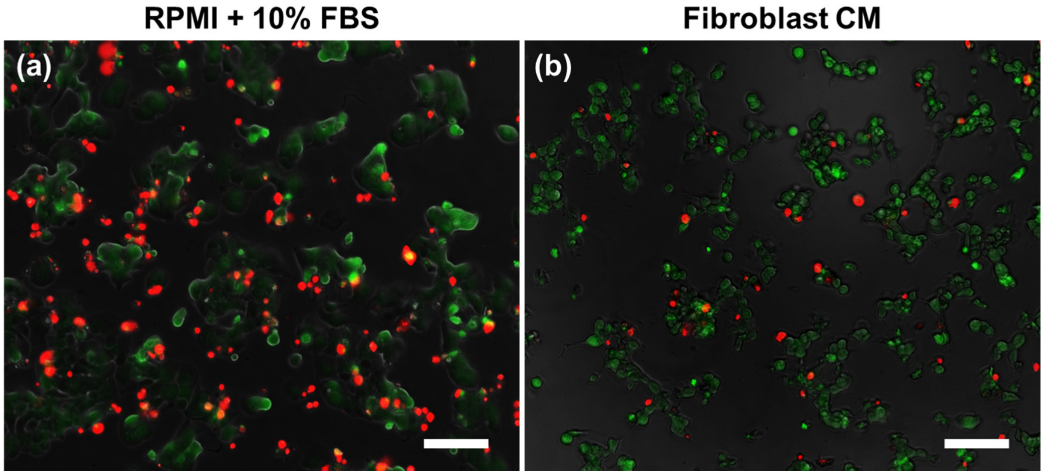

Fig. S7. Cells images after 3 days of 20µM cisplatin treatment: (a) Cells cultured in normal culture media (RPMI+10% FBS) and (b) Cells cultured in fibroblast conditioned media. Using LIVE/DEAD staining, we can visualize cell viability. Fibroblast conditioned media can significantly boost cell viability. (scale bar: 100 µm)

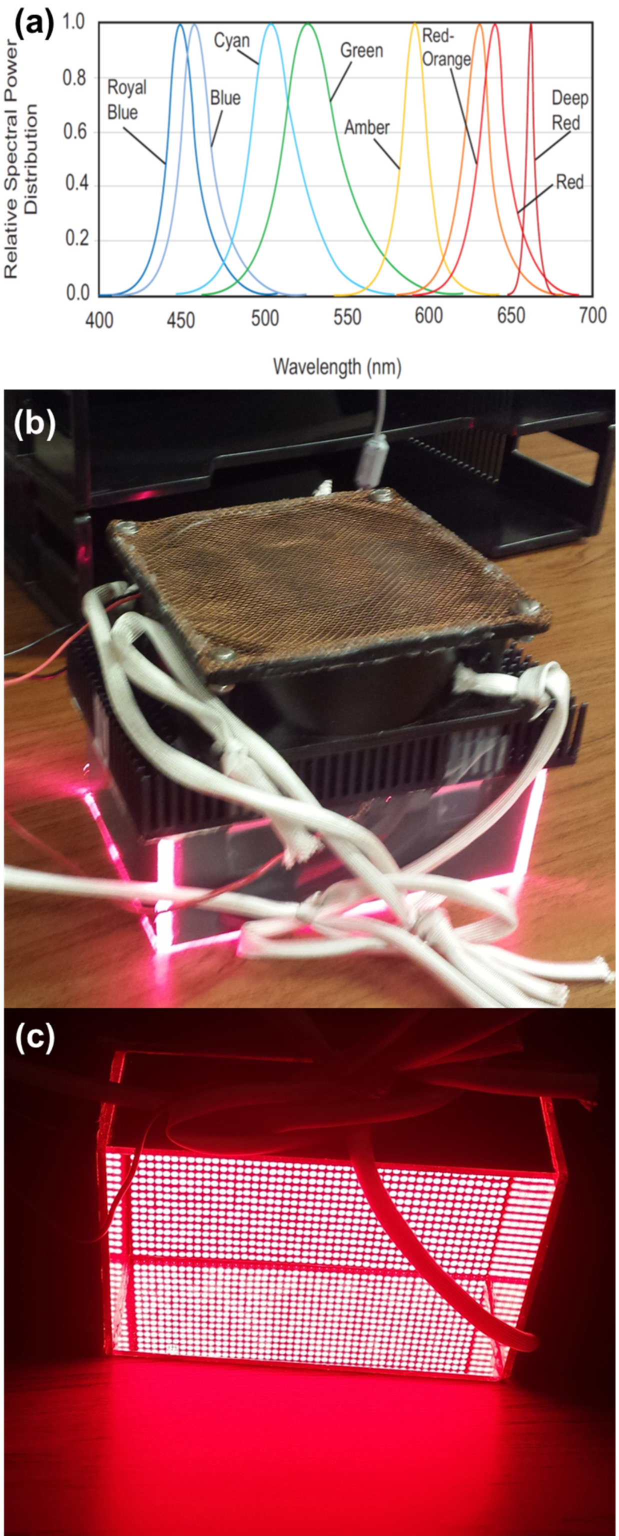

Fig. S8. LED Light source used in PDT. (a) The relative spectral power distribution of the Philips Lumileds LUXEON® Z Red (LXZ1-PD01) LEDs. (b, c) The picture of the assembled LED light source. Illumination intensity is 0.73 J/cm^2/min with uniform brightness (difference < 10%) throughout the whole area.

Cell Viability (%)	2D	3D Large	3D Small
**Methylene Blue Alone (10 μM) Control**	92.7±3.5	93.8±2.8	92.8±4.2
**Light Alone (60 Minutes) Control**	90.2±5.2	94.9±3.6	94.2±4.1
**Sham Control**	97.1±2.4	95.8±3.2	94.3±2.5

Table S1. The cell viability of Methylene Blue alone (10 μM) control, light alone (60 minutes) control, and sham control under 3 cell culture conditions (2D, 3D large sphere, and 3D small sphere).
